# Improvement of Cognitive Function and Interleukin 1 Beta Serum Concentrations Following Cardiac Pacemaker Implantation in Patients with Symptomatic Bradycardia

**DOI:** 10.3390/jpm11080770

**Published:** 2021-08-05

**Authors:** Alexandru Martis, Gabriel Gusetu, Gabriel Cismaru, Dumitru Zdrenghea, Daniel-Corneliu Leucuta, Dana Pop

**Affiliations:** 1 Rehabilitation Hospital Cardiology Department, “Iuliu Hatieganu” University of Medicine and Pharmacy, 400347 Cluj-Napoca, Romania; alexandru_martis2387@yahoo.com (A.M.); gusetu@gmail.com (G.G.); gabi_cismaru@yahoo.com (G.C.); dzdrenghea@yahoo.com (D.Z.); pop67dana@gmail.com (D.P.); 2 Department of Medical Informatics and Biostatistics, “Iuliu Hatieganu” University of Medicine and Pharmacy, 400349 Cluj-Napoca, Romania

**Keywords:** pacemaker, cognitive function, bradycardia, MMSE, trail making test, Interleukin 1 beta

## Abstract

Background and aim: Bradyarrhythmias cause a low cerebral blood flow with secondary neuronal ischemia and cognitive dysfunction. This study aims to assess the effect of cardiac pacemaker implantation (PI) on the cognitive function and inflammatory markers (TNF alpha, IL1β). Material and method: We conducted a prospective observational study on a number of 31 patients with symptomatic bradyarrhythmias. We performed the cognitive function assessment by two tests (Mini-Mental State Examination and Trail Making Test A), cardiac output assessment (echocardiographic), and determination of IL 1β and TNF alpha serum concentrations before pacemaker implantation and after an average period of 42 days from pacemaker implantation.Results: After pacemaker implantation we observed an increase in the cardiac index by 0.71 L/min/m^2^ (*p* < 0.001) and a better scoring in cognitive performance; the mean MMSE score increased by two points (*p* < 0.001), and Trail Making Test A had an improvement of 16 s (*p* < 0.001). Regarding the inflammatory markers, a significant decrease in IL-1β with 8.6 pg/mL (*p* = 0.049) after pacemaker implantation was observed. Additionally, we found statistically significant correlations between IL1β and TNF alpha (positive correlation, *p* = 0.005), between the MMSE and cardiac index (*p* < 0.001), between the Trail Making Test and cardiac index (*p* = 0.001), and between the MMSE and Trail Making Test (*p* = 0.003). Conclusions: Our findings suggest that cardiac pacemaker implantation was associated with improved cognitive function—possibly related to an increased cardiac output and with adecreased serum IL1β concentration in subjects with symptomatic bradycardia.

## 1. Introduction

The prevalence of cognitive decline has continuously increased in the last 3 decades and is currently estimated at 19% among the population over 65 years. Cognitive decline can be seen as a predetermined phase in which the subject still has functional independence. The severity of cognitive decline progresses over time in the absence of a specific treatment; around 120 subjects per 1000 people per year develop dementia [1,2,3].

Vascular dementia is the second cause for dementia after Alzheimer’s disease and is caused by ischemia with different mechanisms of cerebral injury (microembolism, hypercoagulant status, endothelial dysfunction, reduced brain flow) [4,5].

Until now, few studies in the literature have evaluated the influence of pacemakers on improving cognitive parameters in patients with bradyarrhythmias, and we found no reports on the effects of cardiac pacemaker implantation on inflammatory markers (IL 1β TNF alpha). In the last 10 years, some studies have confirmed that the cognitive decline prevalence increases simultaneously with the increase in the number of cardiovascular risk factors (heart failure, diabetes, hypertension, atrial fibrillation) [6].

Data about correlations between the cardiac output and cognitive decline have been highlighted, especially in patients with heart failure. Cognitive decline is influenced by the length of time and the severity of the decreased cardiac output [4].

Interleukin 1β is considered to be involved in the development of dementia, being part of various acute and chronic inflammatory processes, and playing a key role in neuroinflammation and neuronal injury [7,8].

Cardiac output is decreased in bradycardia due to a reduced heart rate, indirectly resulting in a decreased cerebral brain flow. Based on this hypothesis, we investigated whether the treatment of bradyarrhythmia by pacemaker implantation modifies cognitive parameters and inflammatory markers.

## 2. Material and Methods

### 2.1. Study Design and Settings

This is a prospective observational study that included 31 subjects with bradyarrhythmia. We investigated the cognitive function, cardiac output, and inflammatory markers, which were assessed before and after implantation of the cardiac pacemaker at an average period of 42 days.

Patients were hospitalized in the Cluj-Napoca Rehabilitation Hospital, Cardiology Department, Romania, all of them being diagnosed with bradyarrhythmia by EKG or Holter EKG. Their main complaints were syncope, dizziness, loss of balance, visual disturbances, and decreased exercise tolerance. Indication for pacemaker implantation (PI) was following current guidelines (Cardiac Pacing and Cardiac Resynchronization Therapy Guidelines issued by the European Society of Cardiology).

Evaluation of the patients was performed before and after the PI and consisted of 3 parts:

(1) The cognitive assessment, for which we used the psychometric scales Mini-Mental State Examination and Trail Making Test A.
(2) Calculation of cardiac output estimated by echocardiography.
(3) Evaluation of serum concentration of Interleukin 1β and TNF alpha.

All cardiac pacemakers implanted were rate-adaptive with pacing rates between 60 beats/min and 130 beats/min depending on the mobilization of the patient.

### 2.2. Participants

Our study included patients with symptomatic bradyarrhythmia with an indication for pacemaker implantation according to ESC guidelines, at least 8 years of school education, and ejection fraction over 40%.

Exclusion criteria were: dementia, severe valvulopathies, heart failure NYHA III or IV, history of stroke, severe carotid stenosis, advanced chronic renal failure, sensory deficits that made cognitive examination impossible, psychiatric disorders (dementia, alcoholism), or any form of neurodegenerative disease.

All patients signed the informed consent, and the study protocol was approved by the institution’s Ethics and Research Committee (approval registration number 2605/04.04.2018).

### 2.3. Evaluation of Parameters

By echocardiography, we obtained cardiac index (CI) by measuring the left ventricle outflow tract (LVOT) diameter, LVOT velocity time integral (VTI) using pulse-wave Doppler, and heart rate, applying a specific formula adjusted to the body surface. Results of this non-invasive method have a strong correlation with measurements obtained by invasive methods such as pulmonary artery catheter thermodilution technique [9]. CI values below 1.9 L/min/m² define the low cardiac output [10].

Blood samples for IL-1β and TNF alpha were collected in the morning, then rested for 15 min in specific vacutainers. The samples arrived within 30 min at the Hospital Laboratory, where they were centrifuged, followed by freezing the serum at −80 degrees Celsius. Serum level was measured by ELISA using Elisa Kit with Pre-Coated Plates Legend Max Lot No. B238395, the limit of detection being 0.01 pg/mL for IL-1β and 0.1 pg/mL for TNF alpha.

Cognitive tests were performed in a quiet environment, without noise or other disruptive factors. Cognitive function was assessed by a single investigator throughout the study. The Mini-Mental State Examination (MMSE) is a questionnaire that is extensively used in clinical and research settings to measure cognitive impairment (any impairment in perceptual, learning, memory, linguistic, or thinking abilities). It contains a set of questions that assess the patient’s ability for temporospatial orientation, attention, memory, language, and calculation, with a maximum score of 30 points. Scores below 25 points define cognitive impairment with a sensitivity of 87% and specificity of 82% [11]. The Trail Making Test A is a neuropsychological test that evaluates visual attention and executive function, processing speed, and mental flexibility. The test requires a subject to connect in the shortest possible time a sequence of 25 consecutive targets on a sheet of paper. The normal range to complete the test varies according to the patient’s age, being on average between 30 and 38 s, a value over 78 s is considered being abnormal [12]. TMT A exhibits a sensitivity of 77% and specificity of 92% in screening for cognitive impairment [13].

The reason why we chose to evaluate the cognitive function with MMSE and TMT A tests was that they are simple to apply by any practitioner, with good specificity and sensitivity (>80%) [14]. Another reason is that these tests are easy to understand by persons with low educational levels. In our study, the majority of them had between 8 and 12 years of education.

The assessments described above were performed before PI and repeated at a mean period of 42 days after the procedure.

### 2.4. Statistical Analysis

Categorical data were presented as absolute and relative frequencies. Quantitative data were presented as means and standard deviations when normally distributed, and as medians and interquartile ranges when non-normally distributed. Comparisons for repeated measures of non-normally distributed quantitative data were performed with the Wilcoxon test for repeated measures. The correlation coefficient for repeated measures, along with 95% bootstrapped confidence intervals and *p*-values, were computed with rmcorr package [15].

For all statistical tests, two-tailed *p*-values were computed, and a 0.05 level of significance was used.

All analyses were computed in R software (The R Foundation for Statistical Computing, Vienna, Austria), version 4.0.3 [16].

## 3. Results

Baseline characteristics are shown in Table 1. The mean age was 70 years, the majority being male (61%). Hypertension was the most common risk factor, encountered in 87% of participants. Body mass index (BMI) was 28 kg/m^2^, 80% of the subjects being with a high BMI. 

The mean follow-up time was 42 days; 32% of subjects had at least one episode of syncope. A high degree atrioventricular block was the most common indication for pacemaker implantation, followed by atrial fibrillation with a slow ventricular rate. The most commonly implanted type of pacemaker was the VVIR. 

Before the pacemaker implantation (PI), 25% of the subjects had a mild cognitive deficit (MMSE < 25 points). After PI, only 6.4% remained with an MMSE score suggestive for cognitive impairment. Regarding the TMT A score, 51% of the subjects had abnormal values (TMT A > 78 s), after PI, only 38% remained with abnormal values.

The mean pacing capture was 89.5%. At the follow-up examination, 30 patients presented the parameters of pacemaker function within normal limits. One subject presented a pacing deficit that appeared on day 7 after the implantation procedure, presenting 1% pacing capture from total heartbeats. This patient did not show any improvement of the cognitive function (MMSE score 25 points, while the TMT A test showed a worsening result from 154 s to 170 s).

After the implantation of the cardiac pacemaker, an increase in mean HR from 51 b/min to 65 b/min was observed with a concomitant increase in cardiac index by 0.71 L/min/m^2^. Prior to implantation, 90.3% of subjects had a cardiac index (CI) <1.9 L/min/m^2^; after implantation, only 45.2% of subjects remained with a low CI (Table 2).

**Figure 1:**
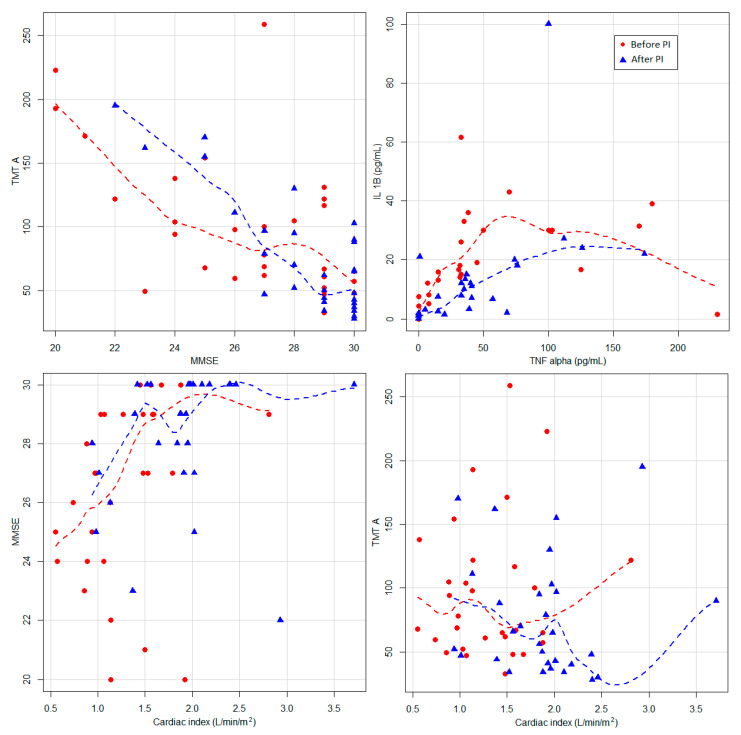
Relation between cardiac index and MMSE and TMT A. The lines represent locally weighted scatterplot smoothing curves. MMSE—Mini Mental State Examination; TMT A—Trail Making Test A; PI—pacemaker implantation; TNF alpha—Tumor Necrosis Factor Alpha; IL-1β—Interleukin 1 Beta.

Cognitive assessment after PI found better MMSE scores with a mean increase from 27 points to 29 points. Additionally, the time to complete the TMT A test was reduced by a mean of 16 s (Table 2). At the same time, a positive correlation was observed between the increase in CO and the improvement of the MMSE score, respectively, the increase in CI and the decrease in TMT A values, as well as a correlation between the improvement of the MMSE score and TMT A values (Table 3, Figure 1).

Serum IL 1β concentrations decreased after PI by a mean of 8.6 pg/mL, resulting with a statistical significance (*p* = 0.049) (Table 2). We did not find any statistically significant differences regarding TNF alpha concentrations, but we noticed a positive correlation between concentrations of IL1β and TNF alpha (*p* = 0.005) (Figure 1).

## 4. Discussion

The results of this prospective study suggest that, following pacemaker implantation, there is an increase in cardiac output, improvements of cognitive function, and a decrease in IL 1β serum concentrations. We also found a positive correlation between an increased cardiac output and improvement of cognitive parameters, as well as a correlation between IL1β and TNF alpha. The data suggest that symptomatic bradycardia causes low cerebral perfusion, cerebral self-regulation being insufficient in long-term chronic hypoperfusion [18]. Chronic hypoperfusion consequences in patients with bradycardia were impaired cognitive function and the activation of the inflammatory immune system. Treatment of bradyarrhythmia by PI not only determines an increased heart rate, but also improves cognitive function and decreases serum IL1β levels.

Data from the literature are few. There are several studies that highlight the presence of a cognitive decline in cardiac pacemaker patients without objectifying whether or not pacemaker implantation brings improvements in cognitive functions [19,20]. Our study is the first study that evaluates IL-1β serum concentrations related to cardiac pacemaker implantation.

Efimova et al. assessed cognitive function after pacemaker implantation in a small group (17 patients), having a low CO caused by atrial fibrillation with a high ventricular rate and which required atrioventricular node ablation and PI. The study founded favorable results related to the improvement of cerebral perfusion and cognitive performance [21].

Another study by Hiromi Koide et al. on a group of 14 subjects observed an increase in cardiac output by 0.8 L/min, with a similar result to our study where the increase in flow was 1.05 L/min. They also observed improvements in cognitive functions by applying the Wechsler Memory Scale and Block Design Test [22].

A similar study that used TMT found that a low cardiac output <4 L/min was associated with cognitive dysfunction and a poor performance on performing TMT A. In our study, 90% had DC < 4 L/min, and 51% of them had abnormal TMT A values [18].

IL-1β beta normal range in the healthy population is between 0.5 and 12 pg/mL. The mean value in our study was slightly increased (15.45 pg/mL), decreasing at 6.85 pg/mL after pacemaker implantation (*p* = 0.049) [8,23]. The INCHIANTI study, which included the evaluation of 1292 participants of serum IL 1β values, found correlations between cardiac output, cognitive function, and IL 1β values; our study indirectly confirming these results [24]. We did not find any studies regarding the impact of PI on Interleukin 1β serum concentrations. Our study suggests there is a link between the improvement of cerebral perfusion by the PI and inflammatory response. A similar study exists related to the decreased expression of IL-1β-responsible genes in a group of patients with cardiac resynchronization therapy responders [23].

The possible confounders for IL-1beta, TNF-alpha were represented by chronic pathologies that influence the inflammatory status: chronic infections, chronic autoimmune disorders, diabetes, neoplasms, but as they were present before and after implantation, their impact was more likely to be low, a higher variation of the inflammatory marker values might have been induced by acute decompensations of these chronic conditions, and some of these might even not be identified. The implantation of the pacemaker comes with the possible risk of pocket infections, or an inflammatory response to the device, which would increase the values of inflammatory markers. Possible confounders for psychological status (MMSE, TMT A) were represented: age, history of hypertension, heart failure, atrial fibrillation, alcohol consumption, hypoxemia secondary to severe chronic obstructive pulmonary disease, sleep apnea syndrome, asthma, syncope, stroke, neurodegenerative disease (dementia), and psychiatric disorders. Some of these were excluded during the patient selection process. The majority are chronic conditions, that were present both before and after pacemaker implantation; thus, likely having a low effect on the measured variables.

There were some limitations of this study. The most important was the fact that the study design was observational, non-randomized, and without a control group, which implies that confounding could be an important issue. This type of methodology allowed us only to control the possible confounders by excluding some of them during the selection of the subjects for the study (dementia, psychiatric disorders (dementia, alcoholism), or any form of neurodegenerative disease; severe valvopathies, heart failure NYHA III or IV, history of stroke, severe carotid stenosis, advanced chronic renal failure). The before–after design that focused the comparisons on the intraindividual scope limited the stable chronic conditions effects on the measured outcomes. However, residual confounding or external influences might still be an issue. The before–after design can lead to internal selection bias, can be subject to secular trends (that cannot be identified), or the regression to the mean effect, or other external influences on the study participants, including lifestyle changes and the Hawthorne effect. Another limitation of the study was its small sample size. Nevertheless, this study had one of the largest sample sizes compared to other studies in the literature [22,25,26,27,28].

## 5. Conclusions

Our study observed that after pacemaker implantation in patients with symptomatic bradycardia, there was a statistically significant increase in the cardiac output, an improvement of cognitive functions (higher MMSE scores, shorter Trail Making Test A time), and a decrease in serum IL 1β concentrations. We also observed a statistically significant direct proportional relation between the cardiac index and positive results of cognitive tests (TMT A, MMSE).

## Figures and Tables

**Table 1:** Baseline characteristics.

Characteristic	Number (%) (*n* = 31)/Median(IQR)
Age (years), mean (SD)	70.87 (6.95)
Sex (F vs. M)	12 (38.71)
Follow-up (days), median (IQR)	42 (38–44)
BMI (kg/m^2^), median (IQR)	28.68 (24.58–32.67)
SBP (mmHg), median (IQR)	125 (120–137.5)
DBP (mmHg), median (IQR)	75 (70–80)
Blood tests	
Uric Acid, median (IQR)	6.2 (5.24–7.58)
Total Cholesterol, median (IQR)	177 (148.5–200.5)
High Density Cholesterol (mg/dL), median (IQR)	40 (35.5–50.5)
Low Density Cholesterol (mg/dL), median (IQR)	108 (86–125)
Triglycerides (mg/dL) (median (IQR)	103 (88–136)
Creatinine (mg/dL), median (IQR)	0.99 (0.82–1.14)
Blood Sugar (mg/dL), median (IQR)	93 (88–102.75)
Serum Potassium (mmol/L) median (IQR)	4.4 (3.79–4.67)
Serum Sodium (mmol/L), median (IQR)	141 (140–142.5)
Urea (mg/dL), median (IQR)	38 (33.5–46)
Hemoglobin (g/dL), median (IQR)	13.5 (11.95–14.9)
Hematocrit (%), median (IQR)	40 (36.45–44.3)
White Blood Cells, median (IQR)	6.5 (5.8–7.61)
Neutrophils, median (IQR)	61.5 (53.85–66.3)
Erythrocyte Sedimentation Rate, median (IQR)	15.5 (9.25–25.5)
Syncope	10 (32.2)
History of Arterial Hypertension	27 (87)
Diabetes	6 (19.3)
Smoke	9 (29)
Type of arrhythmia	
Atrioventricular block:	11 (35.48)
Sick sinus node syndrome with sinus bradycardia:	7 (22.5)
Tachy–brady syndrome:	3 (9.6)
Atrial fibrillation with low heart rate:	10 (32.2)
* Type of pacemaker implanted	
VVIR	19 (61.2)
DDDR	2 (6.6)
AAIR	10 (32.2)
Pacing (%)	89.5 (74.6–97.6)
Cognitive assessments	
MMSE < 25 points	8 (25.8)
TMT A > 78 s	16 (51.6)

* Modes of cardiac pacing: nomenclature, selection, and indications for permanent cardiac pacing [17]; SD—standard deviation; IQR—interquartile range; MMSE—Mini Mental State Examination; TMT A—Trail Making Test A; BMI—Body Mass Index; SBP—Systolic Blood Pressure; DBP—Diastolic Blood Pressure.

**Table 2:** Characteristics in dynamics.

Variables	Before PI	After PI	Absolute Difference (95% CI)	*p*-Value
Heart Rate (beats/min), median (IQR)	51 (47.5–60)	65 (60–70)	14 (12.5–17.5)	<0.001
Stroke Volume (mL/m^2^), median (IQR)	48.25 (38.93–61.32)	54.48 (46.83–62.83)	6.22 (4.81–12.7)	<0.001
Cardiac Output (L/min), median (IQR)	2.65 (1.86–3.03)	3.71 (2.81–4.09)	1.05 (0.87–1.27)	<0.001
Cardiac Index (L/min/m^2^), median (IQR)	1.21 (0.97–1.58)	1.92 (1.53–2.02)	0.71 (0.46–0.69)	<0.001
Trail Making Test A, median (IQR)	78 (60.23–122)	62 (42–96)	16 (13.75–27.5)	<0.001
Mini Mental State Examination, median (IQR)	27 (24.25–29)	29 (27.25–30)	2 (2–3.5)	<0.001
TNF Alfa (pg/mL), median (IQR)	31.5 (0–48.75)	34 (0.25–53)	2.5 (−38.5–30.5)	0.859
IL-1β (pg/mL, median (IQR)	15.45 (2.42–30)	6.85 (1.12–13.12)	8.6 (0–15.2)	0.049

IQR—interquartile range; CI—confidence interval; PI—Pacemaker implantation; TNF alpha—Tumor Necrosis Factor Alpha; IL-1β—Interleukin 1 Beta.

**Table 3:** Correlation analyses for cognitive function and inflammatory markers.

	TNF Alpha (pg/mL)	IL1β (pg/mL)	MMSE	Cardiac Index (L/min/m^2^)	TMT A
TNF Alpha (pg/mL)	1	0.49 (0.21–0.73), 0.005	−0.27 (−0.55–0.15), 0.139	−0.21 (−0.49–0.24), 0.27	0.12 (−0.19–0.43), 0.524
IL1β (pg/mL)	0.49 (0.23–0.74), 0.005	1	−0.23 (−0.62–0.19), 0.204	−0.22 (−0.59–0.11), 0.235	0.23 (−0.19–0.57), 0.213
MMSE	−0.27 (−0.62–0.13), 0.139	−0.23 (−0.64–0.12), 0.204	1	0.72 (0.6–0.83), <0.001	−0.51 (−0.87–0.34), 0.003
Cardiac Index (L/min/m^2^)	−0.21 (−0.54–0.16), 0.27	−0.22 (−0.58–0.11), 0.235	0.72 (0.61–0.82), <0.001	1	−0.62 (−0.89–0.47), <0.001
TMT A	0.12 (−0.15–0.33), 0.524	0.23 (−0.25–0.57), 0.213	−0.51 (−0.89–0.38), 0.003	−0.62 (−0.87–0.52), <0.001	1

IQR—interquartile range; CI—confidence interval; PI—pacemaker implantation; TNF alpha—Tumor Necrosis Factor Alpha; IL-1β—Interleukin 1 Beta; MMSE—Mini Mental State Examination; TMT A—Trail Making Test A; CO—Cardiac Output. Data are presented as correlation coefficient (95% bootstrapped confidence interval), *p*-value.

## Data Availability

Data available on request due to restrictions e.g., privacy and ethical.
